# In Field Fruit Sizing Using A Smart Phone Application

**DOI:** 10.3390/s18103331

**Published:** 2018-10-05

**Authors:** Zhenglin Wang, Anand Koirala, Kerry Walsh, Nicholas Anderson, Brijesh Verma

**Affiliations:** 1 Centre for Intelligent Systems, Central Queensland University, Rockhampton, Queensland 4701, Australia; z.wang@cqu.edu.au (Z.W.); b.verma@cqu.edu.au (B.V.); 2 Institute of Future Farming Systems, Central Queensland University, Rockhampton, Queensland 4701, Australia; a.koirala@cqu.edu.au (A.K.); nicholas.anderson@cqumail.com (N.A.)

**Keywords:** fruit sizing, image processing, Android, mobile phone, OpenCV

## Abstract

In field (on tree) fruit sizing has value in assessing crop health and for yield estimation. As the mobile phone is a sensor and communication rich device carried by almost all farm staff, an Android application (“FruitSize”) was developed for measurement of fruit size in field using the phone camera, with a typical assessment rate of 240 fruit per hour achieved. The application was based on imaging of fruit against a backboard with a scale using a mobile phone, with operational limits set on camera to object plane angle and camera to object distance. Image processing and object segmentation techniques available in the OpenCV library were used to segment the fruit from background in images to obtain fruit sizes. Phone camera parameters were accessed to allow calculation of fruit size, with camera to fruit perimeter distance obtained from fruit allometric relationships between fruit thickness and width. Phone geolocation data was also accessed, allowing for mapping fruits of data. Under controlled lighting, RMSEs of 3.4, 3.8, 2.4, and 2.0 mm were achieved in estimation of avocado, mandarin, navel orange, and apple fruit diameter, respectively. For mango fruit, RMSEs of 5.3 and 3.7 mm were achieved on length and width, benchmarked to manual caliper measurements, under controlled lighting, and RMSEs of 5.5 and 4.6 mm were obtained in-field under ambient lighting.

## 1. Introduction

In-field sizing of fruit on tree can be used to provide information on rate of fruit growth and thus timing of harvest maturity, for estimation of pack-house packaging resource requirement and to inform marketing decisions [1]. For example, Zude et al. [2] reported on use of manual in-field fruit sizing for the estimation of cherry fruit harvest maturity. Wang et al. [3] reported in-field sizing of mango fruit to gauge packing tray size requirements for the crop.

Current best practice for estimation of fruit size in orchard involves measurement by calipers, fruit sizing rings or circumference tapes. These measures require a certain level of operator attention, particularly when manual transcription of results is required. Relatively low cost (< USD $1000) digital fruit sizers with data logger functionality are available (e.g., from Guss Manufacturing, Strand, South Africa), with transfer of data to a connected lightweight computer or tablet possible, e.g., as utilised in studies by References [4,5]. However, the available tools lack inbuilt geolocation and wireless communications capacity, and inherently rely on the operator purchasing and carrying specialist hardware.

The use of machine vision for fruit sizing on packing lines began in the 1970’s and is now well established [6,7]. In this application, a light box installed over the conveyor allows uniform lighting, a background of contrasting colour, fixed camera to fruit distance and angle, and use of multiple cameras. For example, Spreer and Müller [8] used two colour cameras with a fixed camera-to-fruit distance to assess mango fruit length, width, and thickness in a pack line scenario. Machine vision can also be applied to in-field estimation of fruit size, given knowledge of camera to object (fruit) distance. This can be achieved by inclusion of a scale bar in the object plane, e.g., as done by Reference [9]. To avoid this requirement, Regunathan and Lee [10] employed four ultrasonic depth sensors, however, the method produced an average distance to canopy rather than distance to individual fruit. In consequence the root mean square error (RMSE) on the size estimate of citrus fruit on tree was 19 mm (calculated from neural network estimate in Table 2 of [10]). The use of a RGB-D (depth) camera was suggested by Reference [11] for the measurement of object volume (i.e., boxes), while Kongsro [12] proposed use of the low cost Kinect RGB-D camera (Microsoft, Redmond, WA, USA) in the estimation of pig weight. In a parallel work, our group has used a Microsoft Kinect V2 RGB-D camera mounted to a farm vehicle to measure mango fruit length and width of non-occluded fruit on tree, with RMSE of 4.9 and 4.3 mm achieved, respectively [3]. The disadvantage of this system is the requirement for relatively bulky, specialist equipment.

The ‘smartphone’ is now a ubiquitous handheld communication and computing device, effectively carried at all times by all farm staff. Seifert et al. [13] used the data storage and communication capability of the smartphone for management of manual measurements of cherry fruit dimensions. However, these devices possess cameras, geolocation capability, capacity for real time image processing and the ability to transfer data to shared data structures. Therefore, a smartphone application is proposed for the assessment of fruit size, capitalising on existing phone features to achieve a low cost solution in a product carried by the user at all times, and thus accessible for use at any time. Future developments can include image analysis to provide a defect assessment in parallel to sizing estimates. The development of such an application is relatively straightforward, with novelty in being the first to achieve this practical solution to in-field fruit measurement, and in addressing the technical challenge of accommodating variation in fruit thickness (i.e., camera to fruit perimeter distance). This approach of in-field fruit sizing using a phone camera is complimentary to our earlier work describing a farm vehicle mounted camera based system [3]. 

## 2. Materials and Methods

A description of operation of the application is given, followed by a description of the experimental characterisation exercises.

### 2.1. The ‘FruitSize’ Application

#### 2.1.1. Description of Overall Operation

An application (‘FruitSize’) was written in Java and C/C++ language using the OpenCV library for image processing, for use on an Android phone (Android 4.4 or above platforms). The application can read parameters such as camera maximum resolution, camera focal length, camera horizontal, and vertical view angles for different phones, and output length and width of an imaged object (fruit). A HTC Desire 820 mobile phone was used for the reported trial work. In this phone the in-built primary camera has a focal length of 3.81 mm, a calculated pixel-bin size of 2.377 μm and 4224 × 3136 pixels (13 Megapixels), with a resolution of 1920 × 1080 pixels utilised as this resolution is supported by most smartphones. A Samsung Galaxy S6 with 16 Megapixel rear camera (Android 7.0) was used to illustrate the use of the application on other mobile phones.

**Figure 1:**
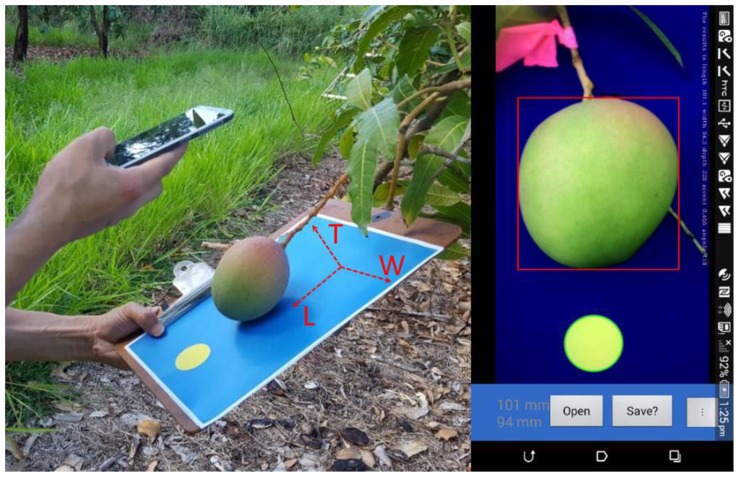
Application scenario (**left**) and main user interface (**right**), illustrating fruit real dimensions of length (L), width (W), and thickness (T).

**Figure 2:**
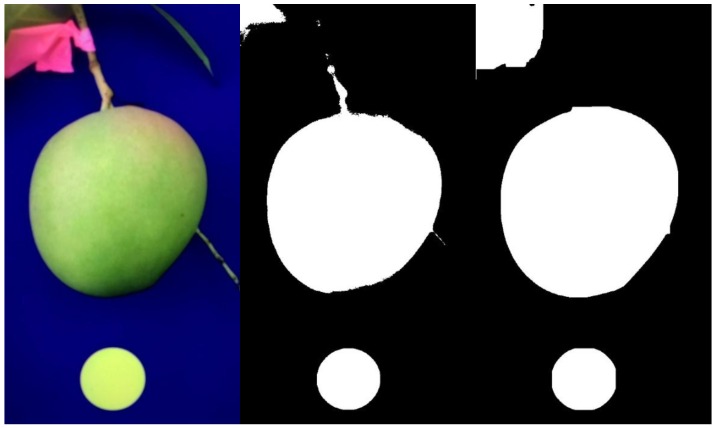
Image processing: image acquisition (**left**); circle identification (**middle**); and fruit segmentation (**right**).

Operation consisted of holding a board with a blue coloured A4 paper sheet behind fruit on tree (Figure 1a). A yellow circle on the backing paper was used as a reference for the estimation of camera to reference plane distance. The blue background was chosen to facilitate segmentation of green, red, and yellow fruit (e.g., Figure 2). The camera to fruit distance was maintained greater than 120 mm (to ensure image coverage of the whole fruit and reference circle) and less than 300 mm (as an operator convenience, with backboard held in one hand and camera in other hand).

#### 2.1.2. Detection of Fruit

**Figure 3:**
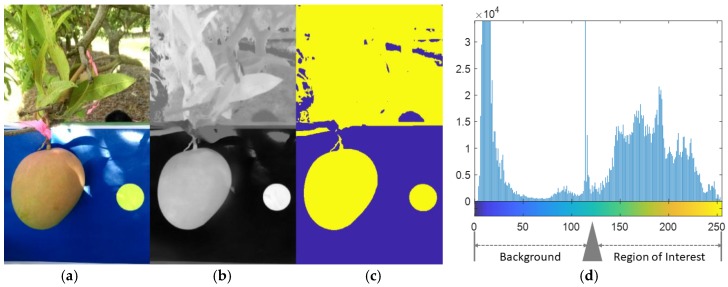
Panels from left to right display (**a**) RGB image of fruit against a blue board and canopy background, in conditions of partial direct sunlight; (**b**) gray scale in the b* channel for the same image, (**c**) the segmented image; and (**d**) a histogram of b* channel values from the image, with the Otsu’s method optimum threshold for separation of background from Region of Interest pixels indicated by the grey arrow.

Two regions in the image are of interest: The reference circle and the fruit. For in-field acquired images, object segmentation is a challenging task because of high variation in lighting condition, including strong shadows on fruits, and variance in fruit traits such as size, shape, colour, and texture. As cameras in different mobile phones differ in colour rendering, a device independent segmentation method was sought. Camera RGB output was converted to XYZ space and then to CIE L*a*b* space using OpenCV functions. A blue background provided contrast to fruit which are typically green, red, or yellow, using the b* channel of CIE L*a*b* colour space. The Otsu’s automatic thresholding [14] was applied on the b* channel of the image to segment fruit and reference object from the background. Otsu’s method seeks a global optimum threshold such that the method can adapt to different light conditions. Thus, thresholding was accurate even with strong direct s4unshine and partial shadowing of the fruit (Figure 3).

Morphological operations were then conducted to remove small objects (peduncle, twigs, leaves, etc.), and a stalk (fruit peduncle) filter based on the priori knowledge that fruit are much larger than the stalks [3], was used to remove the stalks which connect with the fruit. Finally, a dilation operation with a disk structure of one pixel radius was used to generate a smooth curve shape to outline the imaged fruit (examples in Figure 2 and Figure 3). Images were rejected in which the fruit and reference circle made contact.

Finally, object eccentricity and area were used to define the reference circle and the fruit. The findContours function in the OpenCV library was used to find the shapes of all isolated objects and the fitEllipse function was used to judge if the object image shape was close to a circle, using the criterion of eccentricity (ε) calculated from the length (ellipse major axis, *a*) and width (minor axis, *b*) of a bounding box (Equation (1)).

(1) $$\varepsilon =\sqrt{1-{\left(\frac{b}{a}\right)}^{2}}$$

This parameter was estimated for both the reference circle and the fruit. With the phone camera held parallel to the clipboard, the reference circle is imaged as a perfect circle with ε =0.

The eccentricity and area size (in pixels) of the fruit image were used to judge the type of fruit, as introduced in Reference [3]. Briefly, mango and avocado fruit approximate an ellipse shape while apple and citrus fruit approximate a circle shape. This categorisation was used for selection of the appropriate fruit allometric relationship between width and thickness.

#### 2.1.3. Camera to Reference Plane Distance Estimation

Pixel size (p) (in mm) can be estimated from the camera focal length, f (in mm), the total number of horizontal (or vertical) pixels, $W_{m}$, and the camera horizontal (or vertical) angle of field of view, θ (in degrees) (Equation (2)).

(2) $$p=\frac{2f}{W_{m}}\tan\frac{\theta }{2}$$

Usually each cell in a CMOS image sensor is square, so solving p in one dimension is sufficient. The reference circle diameter (in pixels, denoted by ø) was estimated of the segmented image, and its actual size was calculated as product of ø and p. Using the thin lens formula, the distance u (in mm) from the reference plane to the camera was estimated from the known diameter (*D*) of the reference circle (Equation (3)):

(3) $$u=\frac{fD}{øp}$$

#### 2.1.4. Estimating Fruit Dimension

Fruit length, width and thickness were defined in terms of real dimensions ($L_{r}$, $W_{r}$, $T_{r}$, in units of mm) and image dimensions ($L_{i}$, $W_{i}$, in units of pixels). A bounding box was drawn around an object recognised as a fruit. Box vertical and horizontal dimensions represent fruit image length $L_{i}$ and width $W_{i}$, respectively, in units of pixels. The thin lens formula (Equations (4) and (5)) allows calculation of the fruit real length $L_{r}$ and width $W_{r}$ (in mm):

(4) $$L_{r}=\frac{u}{f}pL_{i}$$

(5) $$W_{r}=\frac{u}{f}pW_{i}$$

where u is the distance from the backboard to the camera (in mm).

However, the fruit is a three dimensional object, with the imaged perimeter of the fruit occurring some distance above the reference plane. The reference plane was projected to the fruit perimeter plane using an estimate of the fruit half-thickness. Fruit thickness, $T_{r}$ (in mm), was iteratively estimated using an experimentally determined relationship with fruit width (see later section), as $T_{r}=cW_{r}$ (where c is constant) until $W_{r}$ converged. With each iteration, a revised estimate of $T_{r}$ was used to calculate new values for $L_{r}$ and $W_{r}$ by replacing u with $\left(u-\frac{T_{r}}{2}\right)$. Empirically, this process converged to a stable value of $W_{r}$ within five iterations.

#### 2.1.5. Operating Features

The user requires a simple and interactive interface, with the application reacting to potential errors, e.g., the acquired image is rejected if the camera to object plane angle or the camera to object distance is out of specification. An accepted image is processed and displayed with a green circle overlaying the reference spot and a red bounding box overlaying the target fruit. Time stamp and geolocation data from the smartphone is saved along with fruit size into a CSV file in the phone ‘Download’ folder, with user input required to transfer the file to local computer or cloud server.

### 2.2. Experimental Exercises

#### 2.2.1. Reference Circle Size

A larger reference circle allows for a more accurate assessment of diameter, given limitations of camera resolution. The impact on fruit sizing of size of reference circles was empirically evaluated by mounting the phone on an optical stand with distance to the background varied between 120 and 300 mm in steps of 20 mm as assessed by manual tape measure (accuracy of approximately 1 mm), covering the range of anticipated working distances. Camera to reference plane distance u was calculated from Equation (3) for images acquired of reference circles of a range of diameters (10–70 mm) at each distance.

#### 2.2.2. Camera Tilt

The camera plane should be parallel to the object plane for the most accurate measurement of object size. A circle object will appear as an ellipse in the image if the camera is tilted. To evaluate this source of error on distance estimation, the mobile phone was held on an adjustable optics mount with a fixed distance of 200 mm from camera lens to the backboard, tilted up (+) or down (−) relative to the reference plane.

#### 2.2.3. Fruit Allometrics and Sizing

As a reference measure, fruit length and width were assessed using a digital caliper (DCLR-1205, Clockwise Tools Inc., Palo Alto, CA, USA). Measurement repeatability was assessed as standard deviation (SD) of 20 repeated measurements of a mango fruit. To establish fruit allometrics, the length, width, thickness, and weight of 387 mango fruits of four cultivars (Kensington Pride (KP), Calypso, Honey gold and Keitt) were measured. Fruit were of a range of maturities.

To test the performance of the ‘FruitSize’ application, four exercises were undertaken. Exercise 1: Images were acquired of 20 fruit each of apple (Royal Gala), avocado (Hass), mandarin (Imperial), orange (Navel), and mango (Honey Gold) in a laboratory setting. Exercise 2: Images were acquired of 176 mango (cultivars Kensington Pride and Honey Gold) fruit on tree. Acquisition occurred under a range of lighting conditions, although strong shadows were avoided. Exercise 3: Twenty-one mixed fruit (mandarin, apple and orange) were measured by calipers, and by two operators, using separate phones (Samsung and HTC). Exercise 4: Twenty mango (Kensington Pride) fruit were tagged on tree at stone hardening stage, and assessed weekly over two months, until harvest, using both calipers and the FruitSize application.

## 3. Results and Discussion

### 3.1. Effect of Reference Circle Size on Distance Estimation

Increased reference circle size should result in an improved estimation of the distance from camera to image plane, as the relative uncertainty in the estimation of the diameter of the reference circle is decreased. For example, there is a blur between yellow and blue areas of approximately four pixels at a camera to image plane distance of 200 mm. At this distance, a line of 10 mm is imaged by 80 pixels, and thus an uncertainty of 4 pixels represents 5% of the measurement.

**Figure 4:**
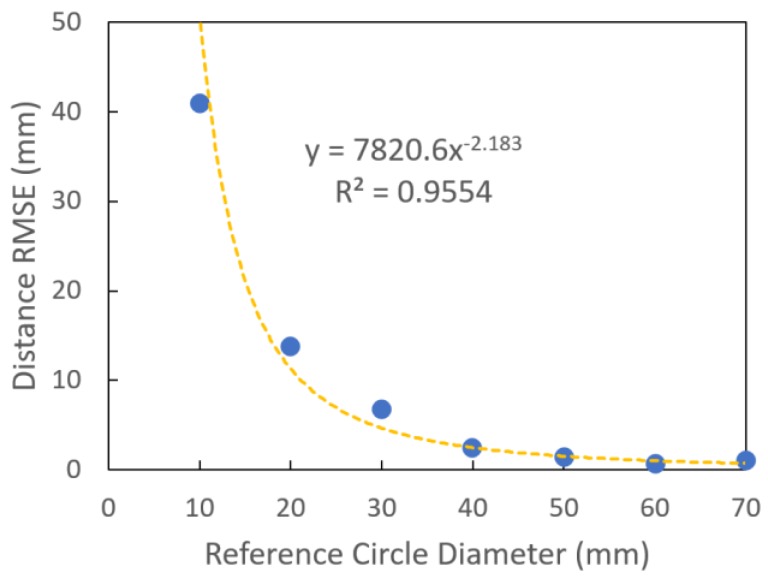
RMSE of camera to object distance estimation (for 10 replicate measurements) as influenced by reference circle size.

The measured RMSE of the camera to reference plane distance estimate decreased as circle size increased, with improvement decreasing for larger diameters (Figure 4). A reference circle size of 40 mm, associated with RMSE of 2.4 mm (Figure 4), was adopted in further work in a compromise between precision and the available space on an A4 sized background. An RMSE of 2.4 mm in distance estimation will introduce a 1.2% error in estimation of fruit size, given a camera to image plane distance of 200 mm (calculation using Equation (3)).

### 3.2. Effect of Camera Tilt Angle

**Figure 5:**
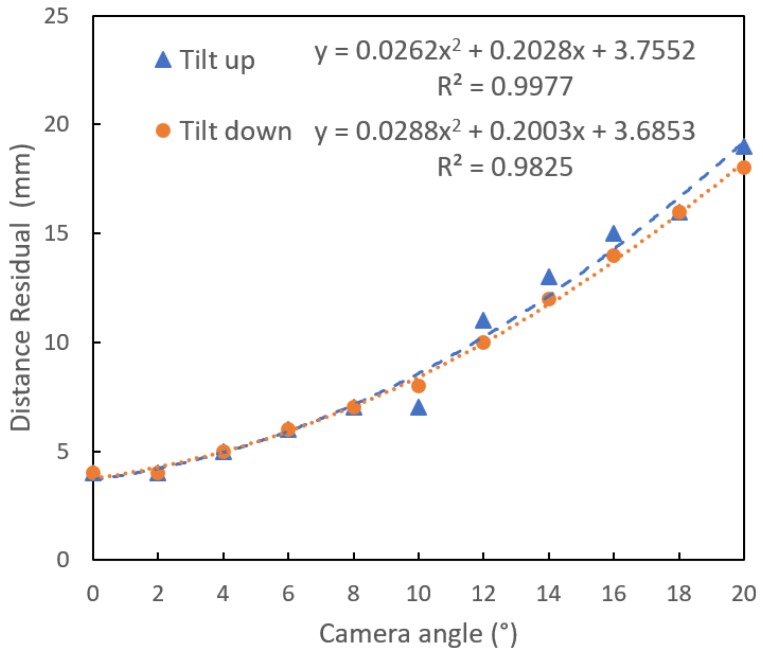
Absolute residual of camera to object distance estimation as influenced by angle between camera (phone major axis) and object planes, for tilt forward (down) and back (up). Camera lens held at a set distance to object plane, while phone body was tilted.

As the angle of the camera to reference plane was increased by tilting the camera long axis, the error of the camera to reference plane distance estimation increased non-linearly (Figure 5) because of distortion of the imaged reference circle. A limit of 14°, associated with an eccentricity of 0.3, was set on the camera-object plane angle for further processing of the image. The RMSE of 12 mm, associated with a 14° tilt, will introduce 6% error on estimation of fruit size for a fruit-to-camera distance of 200 mm (from Equation (3)).

In field practice, there was greater variation in user positioning of the major axis of the phone relative to the backboard than for the minor axis of the phone. Use of the reference circle minor axis (axis parallel to the minor axis of the phone) to estimate camera to reference plane distance therefore achieved better accuracy than use of major axis (data not shown), and therefore this axis was used in scaling the image.

#### Fruit Allometrics

Fruit can be characterized in terms of their lineal dimensions of length, width, and thickness. For some commodities, width and thickness may be similar, and all three parameters are similar for a spherical fruit. An allometric relation between the lineal dimensions of fruit length, width, thickness, and the weight of Chok Annan variety mango fruit was established by Reference [8], and for a range of other cultivars by [15]. Similar allometric relationships between fruit lineal dimensions and weight hold for other fruit. Allometric relations can also be established between lineal parameters, e.g., between length and width.

In holding the backing board behind fruit on a tree, the long axis of the fruit (*L_r_*) naturally aligns with long axis of the backing board. For mango fruit, the wider minor axis (fruit ‘width’, *W_r_*) aligns with the board minor axis, with the dimension of the fruit in the camera to reference plane direction representing fruit ‘thickness’. An allometric relationship established between mango fruit thickness (*T_r_*) and width was stronger than that for thickness and length (Table 1). The relationship *T_r_* = 0.88 × *W_r_* was adopted in the operation of the application. The same relationship was applied to avocado fruit. Mandarin, apple, and citrus were assumed to be spheres, i.e., *T_r_* = *W_r_*.

The RMSE values on length and width estimation of 20 mango fruit were decreased from 8.8 to 5.3 mm and 6.8 to 3.7 mm by the depth correction step. Obviously, the procedure does not give a perfect estimate of camera to fruit perimeter distance, and so represents a source of error in fruit sizing estimation.

### 3.3. Size Estimation Results

Repeatability of the reference (caliper) method was assessed at 1.2 mm for mango fruit (SD of 20 measurements).

Exercise 1: For avocado fruit, RMSE on fruit length and width was 3.4 and 1.6 mm, respectively, while for apple, mandarin and orange diameter, RMSE was 2.0, 3.8 and 2.4 mm, respectively. The RMSE on mango fruit length and width measurements were 5.3 and 3.7 mm, respectively.

**Figure 6:**
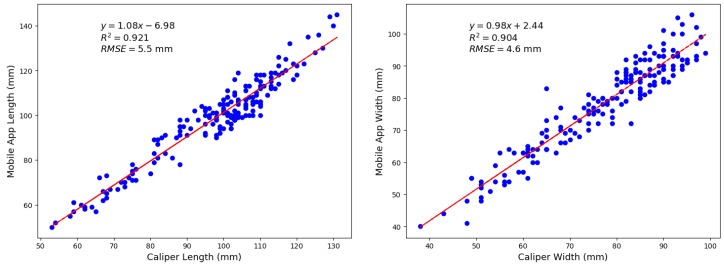
Correlation between mobile application and caliper measurement of fruit length (**left panel**) and width (**right panel**).

Exercise 2: For in orchard measurements of mango fruit on tree (n = 176), the linear correlation of machine vision estimated fruit length and width against caliper measurements was characterised by a R^2^ = 0.921 and 0.904 and RMSE of 5.5 mm and 4.6 mm (bias = +1.0 and +1.1) for length and width estimation, respectively (Figure 6). This RMSE result is equivalent to that achieved in a companion study on in field estimation of mango fruit size using a Kinect time of flight camera [3]. The lower RMSE of the assessments made indoors compared to the field based mango measurements is likely due to better operator performance in terms of holding the phone parallel to the backing board and less segmentation error associated with uniform lighting in an indoor setting.

The bias is attributed to an error either in the lens and pixel specifications accessed from the camera software, or an over-estimate of fruit depth from fruit allometry. However, bias can be empirically corrected in future measurements.

**Figure 7:**
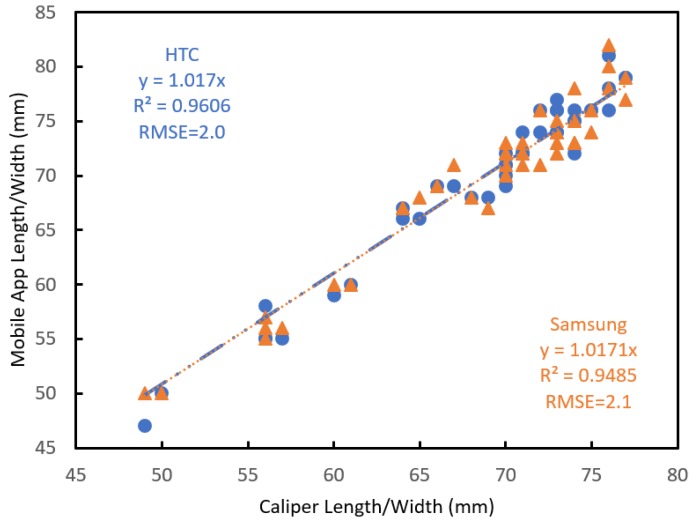
Fruit length and width estimated using the mobile application on two phone types (HTC, Samsung), relative to caliper measurement, for a set of mandarin, orange and apple fruit (n = 21 fruit).

Exercise 3: To illustrate use of the application across mobile phones, two mobile phones were bench marked to caliper measurements, with similar results achieved (RMSE = 2.0 and 2.1 mm for the HTC and Samsung phones, respectively) (Figure 7). The slight difference is attributed to operation error (i.e., different camera tilt angles). The performance consistency of the application on different mobile phones is ascribed to the measurement principle, being based on thin lens theory. A manufacturer/model calibration or bias-correction procedure could increase accuracy, but this approach adds additional complexity (e.g., a list of supported phones).

**Figure 8:**
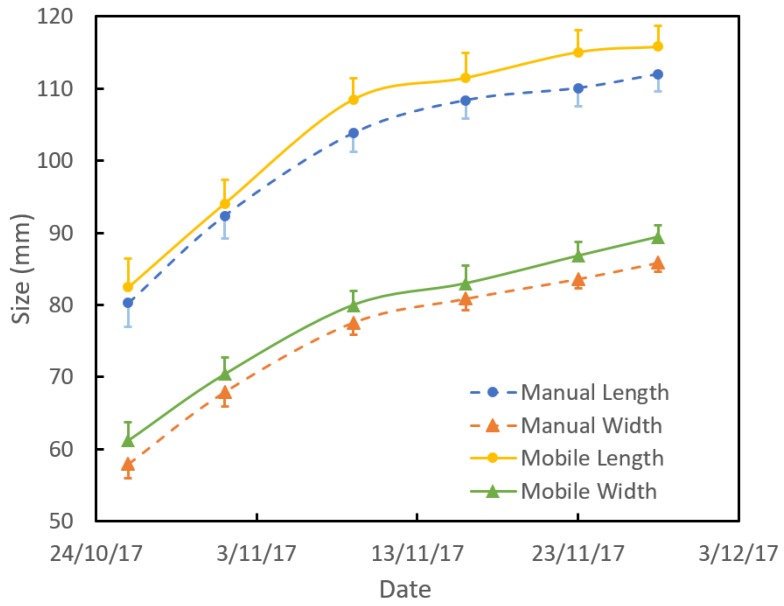
Time course of average mango fruit lineal dimensions (n = 17 fruit) for length (top line pair) and width (bottom line pair), as assessed using calipers (dashed line) or machine vision (FruitSize application; solid line). Error bars represent the standard error of the mean.

Exercise 4: To illustrate use of the application, machine vision and caliper estimates of 20 tagged fruits on tree were acquired weekly over two months (three fruit dropped before the last reading) (Figure 8). The average growth rate over this period was 1.0 mm/day for length and 0.9 mm/day for width, as estimated using either caliper or machine vision. Changes in the rate of growth can be interpreted in terms of growth conditions, e.g., water availability and fruit maturity [15]. Size estimates can be converted to fruit weight estimates based on fruit allometry, as undertaken in the companion study [3].

### 3.4. Fruit Size Application Use

A typical assessment rate of four fruit per minute or 240 fruit per hour was achieved.

The FruitSize application can be used for estimation of population statistics on fruit size, to inform packing and marketing decision making. The required sample for such a task is dependant of the level of population variation. For the population of assessed fruit reported in Figure 5, the standard deviation (SD) on the machine vision length and width estimates was 18.7 mm and 14.2 mm, respectively. Using the t-statistic relationship $n={\left(\frac{t\times SD}{e}\right)}^{2}$ and t = 1.96 (confidence level of 95%, n > 15) and acceptable error of 5 mm, a sample size of 54 (for length) and 31 (for width) samples is warranted. This is a manageable workload, per orchard block.

## 4. Conclusions

In a parallel study we describe in-field fruit sizing based on use of an RGB-D camera mounted to a farm vehicle. The current paper introduces a low-cost solution, based on use of a smartphone. The application utilises the thin lens formulae and a reference marker to estimate camera to background distance, adjusted for camera to mid-fruit plane by an iterative correction from the relationship between fruit length and thickness. The application employs several internal checks of image quality features that impact on size estimation, rejecting images taken outside of the camera to fruit distance of 120 mm to 300 mm, and at a tilt angle between camera plane and object plane of greater than 14 degrees. The eccentricity and area of the imaged fruit was used to allocate the image to a fruit type and associated allometric relationship between fruit width and thickness. Measurement accuracy allowed estimation of fruit growth rate from weekly assessments, and allowed estimation of fruit size distribution. Phone geolocation data allows for field collected data to be automatically assigned to user defined orchard management units, with associated calculations to render weight distribution or projected time to reach a desired size.

Possible improvements include a lightweight frame to hold the camera at a fixed distance and angle relative to the object, with compromise to ease of storage and transport. Use of more than one reference circle (e.g., in the four corners of the backboard) would also be useful in calculating a correction for camera tilt and distance. New phone technology employing a true-depth camera (e.g., iPhone X) or stereo cameras (HTC Evo 3D, iPhone 7 Plus) could be used to obtain the fruit perimeter to camera distance, replacing the need for a reference object. Alternatively, a third-party plug-in ToF-distance measurement device (e.g., Ryobi ES9400) could be used.

The application is available for download at https://www.fruitmaps.com.au.

## Figures and Tables

**Table 1:** Allometric relationships based on linear regression with intercept of zero for fruit real thickness (*T_r_*) to length (*L_r_*) and width (*W_r_*) of mango fruit (n given in brackets).

Cultivar	*T_r_* vs. *W_r_* Relationship		*T_r_* vs. *L_r_* Relationship	
	Slope	R^2^	Slope	R^2^
Honey Gold (42)	0.82	0.72	0.74	0.50
KP (43)	0.87	0.66	0.73	0.47
Calypso (242)	0.90	0.88	0.75	0.65
Keitt (60)	0.82	0.93	0.57	0.92
All	0.88	0.67	0.74	0.60
